# Re-engineering artificial muscle with microhydraulics

**DOI:** 10.1038/micronano.2017.16

**Published:** 2017-06-05

**Authors:** Jakub Kedzierski, Eric Holihan, Rafmag Cabrera, Isaac Weaver

**Affiliations:** 1 Massachusetts Institute of Technology Lincoln Laboratory, Lexington, MA 02420, USA

**Keywords:** artificial muscle, electrowetting, linear actuator, microactuator, microsystem, microhydraulic stepping actuator

## Abstract

We introduce a new type of actuator, the microhydraulic stepping actuator (MSA), which borrows design and operational concepts from biological muscle and stepper motors. MSAs offer a unique combination of power, efficiency, and scalability not easily achievable on the microscale. The actuator works by integrating surface tension forces produced by electrowetting acting on scaled droplets along the length of a thin ribbon. Like muscle, MSAs have liquid and solid functional components and can displace a large fraction of their length. The 100 μm pitch MSA presented here already has an output power density of over 200 W kg^−1^, rivaling the most powerful biological muscles, due to the scaling of surface tension forces, MSA’s power density grows quadratically as its dimensions are reduced.

## Introduction

Significant advances have recently been made in realizing autonomous microsystems, from micro-sized aerial vehicles (μUAVs)^1^, to jumping robots^2^, and even to magnetically controlled systems that can navigate inside the human body^3^. However, there seem to be almost as many modalities for microsystem actuation as there are examples of microsystems. Piezoelectrics^4^, electroactive polymers^5^, complex microelectromechanical (MEMs) structures^6^, and pneumatically flexed elastic membranes^7^ are just a few examples of mechanisms used for actuation. One reason for this divergence is the lack of a truly versatile microactuator. At scales below 1 cm^3^, when inductive motors become inefficient^8^, no type of actuator emerges as the ideal solution. This issue does not occur in biology. Muscle is an excellent actuator on the scale of an ant or an elephant, and its basic structure does not vary significantly. Muscle is versatile, powerful, and efficient. The closest artificial analogs are electroactive polymers, and while these polymers continue to be a promising area of research, they have low output power and some issues with reliability^9^.

**Figure 1:**
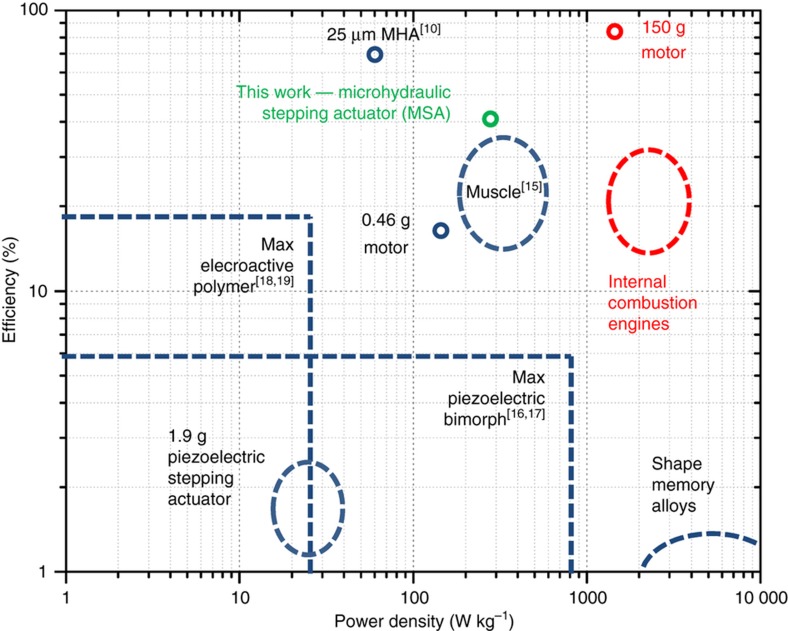
Efficiency and power density relation for various types of microactuators, in blue, and larger actuators, in red. Biological muscle metric is given for maximum power density, not typical motion. Shown in green is the microhydraulic stepping actuator (MSA) from this work. A related microhydraulic electrowetting actuator (MHA) is also included for reference. Efficiency and power density values for commercially available actuators are taken from product datasheets: Internal combustion engine power density is calculated from a Corvette Stingray’s LT1 6.2 L V8, the 150 g motor is the 22ECS60 Ultra EC a brushless DC motor, the 0.46 g motor is the MK04S-24 from Didel SA, shaped memory alloy is the 25 μm diameter Flexinol wire from Dynalloy Inc., the stepping piezoelectric actuator is the LSPA30uXS Linear actuator from Cedret Technologies (attempted to remove payload mass from power density calculation).

In this paper, we describe a new type of actuator, inspired by biological muscle and implemented using concepts from microhydraulics^10^, electrowetting motors^11,12^, and electrowetting microconveyors^13,14^. Our microhydraulic stepping actuators (MSAs) work by integrating force contributions from interfacial tensions along the length of a thin ribbon, much like actin filament in muscle integrates individual stress contributions from myosin heads. MSAs promise a combination of efficiency, power density, and versatility that has not been possible on a microscale (Figure 1 (Refs. 15,16,17, 18,19)). MSAs can also be scaled to higher power, since, like other microhydraulic actuators^10^, their power density scales as the inverse square of the linear scaling dimension.

**Figure 2:**
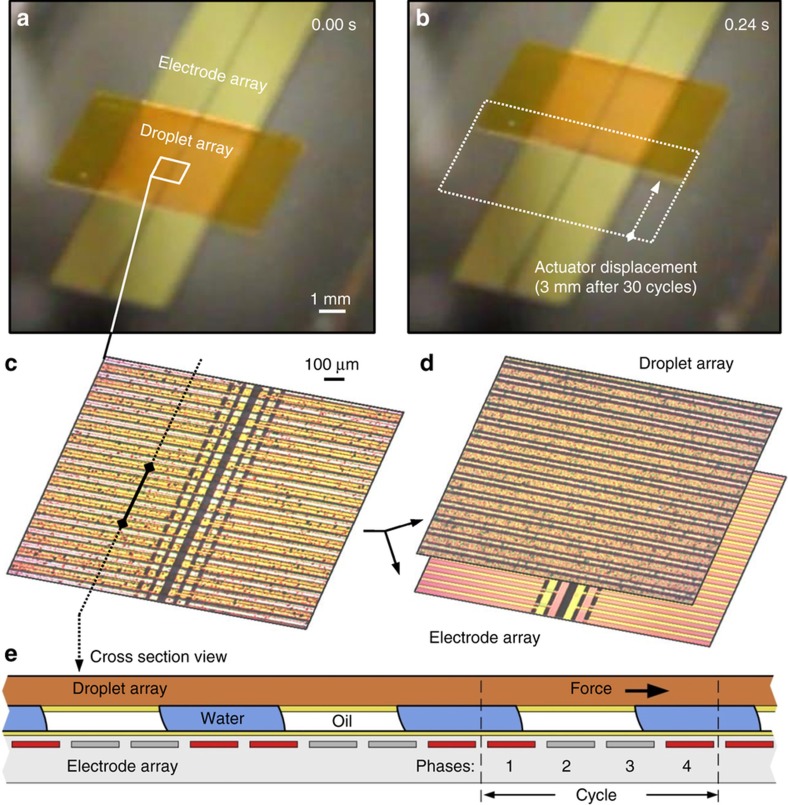
Microhydraulic stepper actuator structure and operation. (**a**) Low-magnification image of the actuator, showing the base electrode array and the rectangular 25-μm-thick polyimide droplet array riding on top. The two arrays are separated by a thin ~15 μm layer of fluid composed of water-miscible droplets and surrounded by oil. The water droplets are attached to the droplet array by patterned hydrophilic regions but are free to slide on the hydrophobic electrode array when the electrodes are off. (**b**) The droplet array displaced by 3 mm in 0.24 s, after 30 cycles of actuation. (**c**) A magnified top–down view of the actuator. The droplet array is sufficiently transparent that the drops between the two arrays as well as the electrodes are visible. (**d**) Separate images of the two arrays, showing the drops on the droplet array and the electrodes on the electrode array. (**e**) A cross section view of the actuator during operation. The electrode array consists of electrodes repeated in cycles; in our design, there are 4 electrode phases per cycle. During operation, electrodes are sequentially turned on, pulling the drops and the droplet array by an electrowetting action. In the current step, the red electrodes (1,4) are on, in the subsequent steps electrodes (1,2), then (2,3), then (3,4) will be on, until the cycle returns to (1,4). Actuation videos are available in the Supplementary Material.

Figure 2 shows the structure and operation of a single-layer linear MSA. The actuator is made from two solid components that move past each other: the electrode array and the droplet array, separated by a fluidic layer. The electrode array consists of a repeating set of electrodes. We used 4 electrodes in a set, or cycle, but any number above 3 works. On top of the electrode array is the droplet array, a thin sheet of plastic or glass with structured semi-cylindrical water-miscible droplets ordered by regions of patterned fluoropolymer. Oil fills the space between the droplets, and the droplet pitch is equal to the electrode cycle pitch: 100 μm. The actuator operates by sequentially energizing the electrodes in a cyclic manner. Each electrode transition creates a step movement in the droplet array by pulling all the attached droplets to the next energized electrode using electrowetting. After 4 steps, the droplet array moves by one droplet pitch, and the electrode sequence can repeat. Figures 2a and b show a 3-mm displacement after 30 cycles. Operation is similar to the operation of a stepper motor, in which cyclic current inputs are used to move a rotor by a fixed number of steps.

Microhydraulic stepping actuators share many advantages of both stepper motors and biological muscle. Similar to stepper motors, MSAs have a high force at low speed and achieve full force at a hold position. They have precise positioning and excellent repeatability. The actuator shown in Figure 2, for example, routinely exhibits less than 5 μm of movement error after many centimeters of travel. The similarities of the actuators to muscle are also significant. Similar to muscle, MSAs are composed of fluid and solid functional components, and with proper materials, MSAs could be made as soft and as light as muscle. However, the similarities are more than skin deep. Because the operation of the actuator is similar to the operation of a muscle, similar trade-offs are possible. For example, the length of a functional muscle unit cell, the sarcomere, can vary. Long sarcomeres, found in crab claws, give high force but lower speed^20^, while shorter sarcomeres, found in vertebrates, give lower force but greater speed. Correspondingly, in MSAs, the number of drops can be varied, even with a fixed cycle pitch. Since each drop contributes a constant force, longer actuators with more drops will have more force. The greater force comes at the expense of speed, since the actuator requires more cycles to displace its total length at a fixed stepping frequency.

## Materials and methods

### Solid component fabrication

Electrode arrays were fabricated on glass wafers (Eagle Glass, Corning, NY, USA; 200 mm, 0.75 mm thick). After a piranha clean, the first metal layer was deposited, patterned, and dry etched (10 nm Ti, 170 nm Al, 10 nm Ti, 25 nm TiN). An inter-layer dielectric was then deposited (300 nm SiO_2_ by plasma enhanced chemical vapor deposition). Next, a second layer of the same metal stack was deposited, patterned, dry etched, and capped with another 300 nm of SiO_2_. Contact openings were defined and dry etched to allow electrical connection to the metal lines. Finally, a 60-nm-thick layer of a fluoropolymer (CYTOP, Asahi Glass Co., Tokyo, Japan) was spun on the wafer and baked at 175 °C for 1 h. The fluoropolymer renders the surface hydrophobic and enables electrowetting. The electrode layout consisted of four 25-μm-wide electrode phases repeated in a cyclic pattern at a cycle pitch of 100 μm. Each electrode was divided in two, down the middle of the array, with a positive potential applied to one side and an equal negative potential applied to the other side when energized. This procedure allowed the droplets to remain at zero potential during electrode activation without a need to ground them electrically.

Droplet arrays were fabricated from two thin substrate variants: sheets of 25-μm-thick polyimide (Kapton HN, American Durafilm, Holliston, MA, USA) and plates of 30-μm-thick glass (Eco30 glass, Schott AG, Mainz, Germany). Thin substrates were processed in 100×100 mm squares by carefully attaching them to stainless-steel frames. Spinning various films onto the thin substrates was aided by a specialized vacuum chuck (Laurell Technologies Corporation, North Wales, PA, USA). Droplet array fabrication started by spinning a 60-nm-thick film of fluoropolymer onto both sides of the thin substrate and baking it at 175 °C for 1 h. Subsequently, one side of the fluoropolymer was photolithographically patterned and dry etched to reveal hydrophilic regions of the substrate amidst the hydrophobic fluoropolymer coating. Hydrophilic droplet regions were 50 μm wide and placed at a droplet pitch of 100 μm. Glass droplet arrays were cut to their final size by careful scribing with a diamond tipped scribe, and the polyimide arrays were cut using a programmable paper cutter (Silhouette Cameo). Active parts of the droplet arrays were 5×5 mm.

### Fluidic actuator assembly

**Figure 3:**
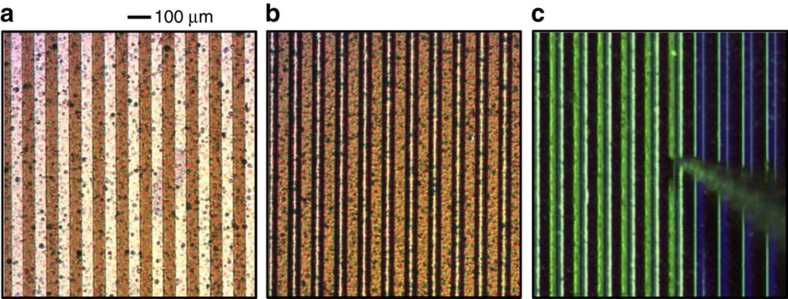
Droplet array assembly. (**a**) An optical image of a dry droplet array after fabrication. Fluoropolymer-covered regions are darker than hydrophilic areas. (**b**) The droplet array after dabbing with triethylene glycol (TEG). Three-μm high droplets of TEG form on the hydrophobic regions. Water drops formed in a similar way last less than a second under laboratory ambient conditions. (**c**) Drop by drop injection with TEG at 0.97 kPa (9 cm TEG) to a TEG drop height of 6 μm.

**Figure 4:**
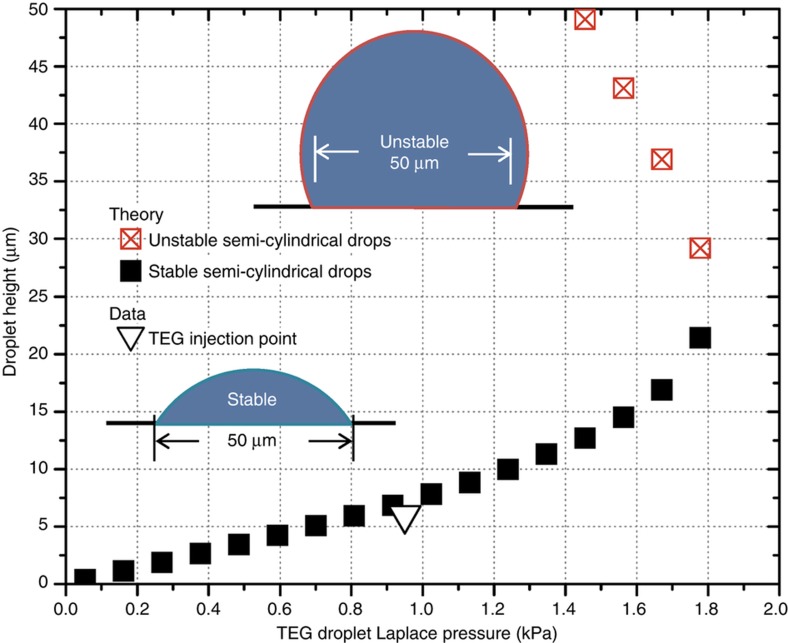
Relation of internal droplet Laplace pressure (surface tension divided by the radius) for a semi-cylindrical segment droplet of triethylene glycol (TEG) in air. The graph shows the 0.97 kPa injection point that sets the TEG drop height prior to water absorption. Some small amount of water already exists in the TEG because of the ambient laboratory humidity. The plot also shows the point of maximum internal pressure, which occurs at a minimum droplet radius and height of 25 μm, half of the 50-μm chord length. Above this height, any semi-cylindrical drop formed on the array, regardless of surface tension, is unstable and will quickly collapse to a semi-spherical profile.

**Figure 5:**
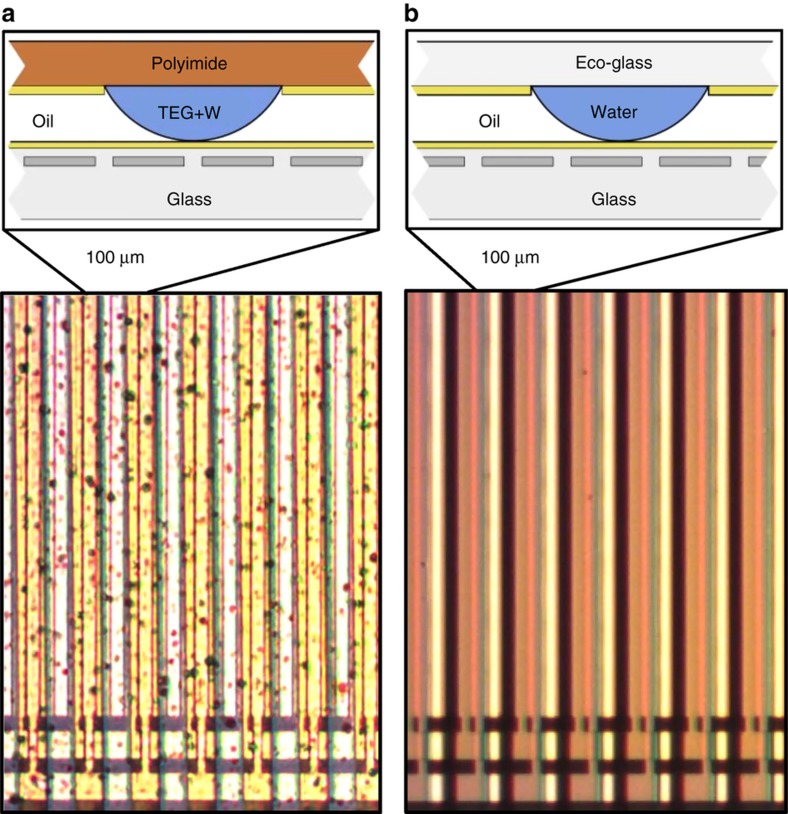
An optical image of finished MSAs with a (**a**) 25-μm-thick polyimide droplet array, and a (**b**) 30-μm eco-glass droplet array. Drop pitch and cycle pitch is 100 μm, as shown in the insert. Particles seen in the polyimide droplet array are native to the polyimide and do not significantly affect its roughness.

Following solid component fabrication, the fluidic parts of the actuator were added, and the actuator was assembled by combining the two arrays. The most challenging part of this process was obtaining small, uniform, semi-cylindrical water-miscible drops on the droplet array. Two techniques were developed, one for each type of droplet array. For eco-glass arrays, water was selectively condensed onto the hydrophilic regions by cooling it to 2 °C below the dew point (11 °C in our laboratory). Once water droplets achieved a height of 18 μm, the droplet array was wicked onto the electrode array using a 2-μL drop of dodecane (oil phase) positioned on the electrode array surface, thus mating the two arrays and finishing the assembly. Another method for droplet formation was used on the polyimide droplet array. First, the array was wetted with triethylene glycol (TEG), resulting in the formation of shallow (~3 μm tall) TEG droplets. Next, each drop was injected with additional TEG at a fixed Laplace pressure (0.97 kPa) using a micropipette with a 30-μm-diameter opening; Figures 3 and 4. This process equalized the size of all drops to a height of 6 μm. After injection, TEG droplets were exposed to 100% humidity at room temperature and absorbed water, growing to a height of 18 μm. The hydroscopic water absorption is somewhat self-limiting and was timed at 6 min. Immediately afterwards, still in 100% humidity, the electrode array with 2 μL of oil was used to wick the droplet array, mating the two arrays and finishing the assembly. Figure 5 shows the finished glass and polyimide based actuators.

Correctly tuning the final heights of the water droplets is critical to actuator operation. If droplets are of different heights, shorter droplets may not actuate evenly when voltage is applied. Since the distance between the two arrays is set by the droplets, a higher droplet height increases array separation and reduces the viscous resistance of the actuator. However, a drop height limit exists. At a height of half the hydrophilic region width, or 25 μm in our case, the droplets become unstable and collapse from semi-cylindrical to semi-spherical. The point of instability corresponds to the maximum internal drop Laplace pressure. Figure 4 shows how Laplace pressure, height, profile, and stability in a TEG drop are related, although the height limit is independent of fluid composition. It is important to stay below this limit during assembly and operation. At the end of MSA assembly procedures, the internal droplet pressures relative to the oil phase were ~1.1 kPa for eco-glass droplet arrays and 0.57 kPa for the polyimide arrays. The difference is due to changes in surface tension caused by TEG.

## Results

**Figure 6:**
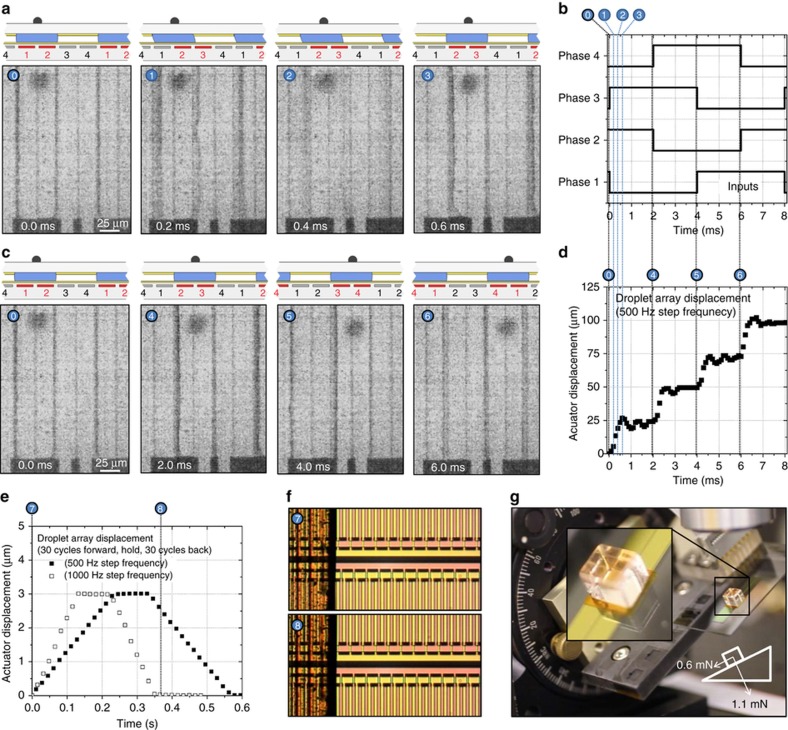
Microhydraulic stepping actuator (MSA) actuation results. (**a**) The actuation frames from a high speed (10 000 frames per s) camera, showing an eco-glass droplet array displacing one step in 0.6 ms. (**c**) Frames from 4 subsequent steps, or one cycle, from an actuation of an eco-glass droplet array at a step frequency of 500 Hz. (**b**) and (**d**) The electrode phase input voltages and the digitized droplet array displacement, respectively, for the actuation shown in **a** and **c**. Also shown are the time slices corresponding to the captured frames. **d** clearly shows that the droplet array moves in discrete steps and undergoes underdamped oscillations around each step position. Inertial effects are largely due to the mass of the droplet array, while the damping is caused by viscous effects of the fluids, the spring action is caused by surface tension of the drops. (**e**) Displays the macrodisplacement of a polyimide droplet array. The droplet array is displaced 30 cycles forward, held for 0.1 s, and displaced 30 cycles back to its original starting position. Displacement occurs twice as fast at a 1000 Hz step frequency as at 500 Hz. (**f**) The optical image of the edge of the droplet array before and after the 1000 Hz actuation shown in **e**. Due to the digital control of the actuation, the droplet array returns to its original position within a couple of microns, even after displacing 3 mm. (**g**) A simple example of the actuator performing work against an external load. A 0.13 g block of plastic is placed on the microgram droplet array, and the entire actuator is rotated to incline at 30° to the horizontal. The actuator can still controllably actuate in both directions against and with the load (0.6 mN). It can also resist a significant normal force (1.1 mN). Videos of actuations shown in this figure are available in the Supplementary Material.

Actuators were driven at ±30 and ±40 V for phases 1, 3 (second fabricated metal layer) and 2, 4 (first metal layer), respectively, to account for the difference in dielectric thickness between metal levels. Voltage and timing were provided by a LabVIEW controller. Prior to movement, phases 1 and 2 were activated to execute a hold step, which acts to pull the water droplets into close contact with the electrode array. The hold step also causes the two parts to self-align^21^, both in rotation and translation. Actuation proceeds after the hold step by activating the electrodes in a cyclic manner. In 4-phase actuators, four steps are executed per cycle, as shown in Figures 6a–d. The cycle can be repeated an arbitrary number of times, as shown in Figures 6e-g. For backward motion, the step sequence in the cycle is simply reversed. Actuator stepping motion is discrete; once the electrodes change state, the droplet array moves one electrode width, or 25 μm. Actuation details are captured by a high-speed camera in Figures 6a–d. The droplet array moves into the next step position in less than a millisecond, achieving velocities as high as 80 mm s^−1^ and accelerations of 0.26 km s^−2^. At each step, the droplet array undergoes underdamped oscillations due to the inertia of the droplet array and the elasticity of the droplet interfacial tensions. Using the values of maximum velocity, acceleration, and the droplet array mass, the actuating force and maximum power density can be calculated to be 0.69 mN and 278 W kg^−1^, respectively. For reference, powerful biological muscles such as the hummingbird flight muscle have a maximum power of 309 W kg^−1 ^Ref.(22). The actuating force at maximum power is lower than the blocked force that was measured during a hold step to be 2.8 mN. Actuating force is calculated using Newton’s Law as *m*×*a*, using the glass droplet array mass of 2.6 mg. Power is calculated as *F*×*v*, and normalized by the functional actuator mass, that of the fluids, dielectric, and electrodes. The blocked force is measured using a small spring as the minimum force required to dislodge the stationary droplet array in the direction of motion during a hold step. Figure 6e shows the macro motion of the MSA, when a polyimide droplet array is actuated 30 cycles forward and 30 cycles back, at 1000 and 500  Hz stepping frequencies. Figure 6g provides a simple demonstration of the ability of the actuator to perform external work by carrying a 0.13 *g* block of clear plastic up a 30° inclined plane. The actuator can carry >60× its own weight uphill while also resisting forces normal to the array plane. Actuation videos are available in the Supplementary Material.

**Figure 7:**
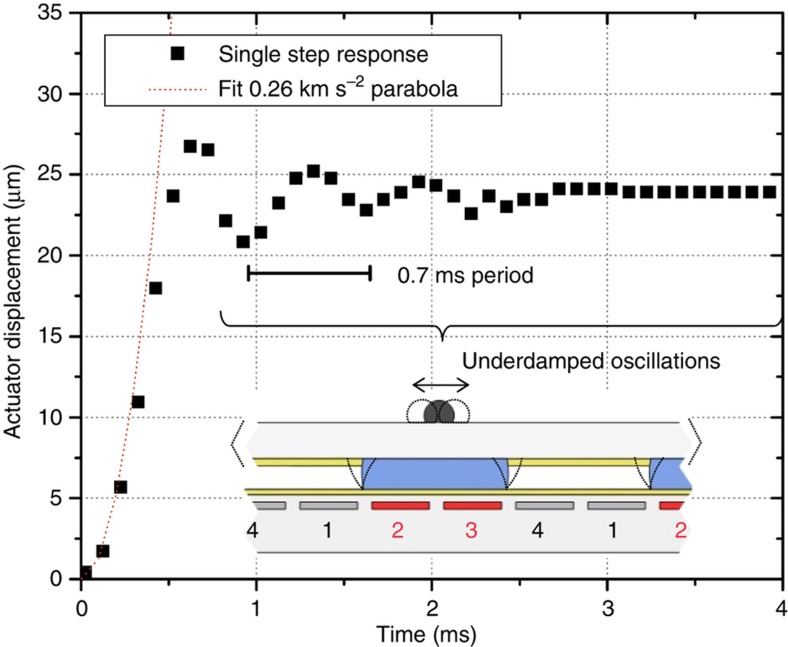
Microhydraulic stepping actuator (MSA) detailed step response. At *t*<0 ms, electrodes 1, 2 are turned on. At *t*=0 ms, electrode 1 is turned off, and electrode 3 is turned on. The actuator displacement is measured as the movement of the droplet array relative to its position at *t*=0 s. Initially, between 0 and 0.4 ms, the droplet array undergoes significant acceleration as it moves to its new equilibrium position at a displacement of 25 μm. The acceleration fits well to a constant of 0.26 km s^−2^, as can be seen by comparing it to a constant acceleration parabola. After 0.5 ms, the droplet array undergoes underdamped oscillations with a period of 0.7 ms.

Further insight into actuator behavior can be obtained by looking at the details of the step response. Figure 7 shows displacement for a glass droplet array during a single step. At *t*=0 s, electrode 1 turns off and electrode 3 turns on. Therefore, the droplet array moves one 25-μm step. During the initial part of the movement, acceleration is nearly constant at 0.26 km s^−2^, as can be seen by the parabolic fit of the data between 0 and 0.4 ms. At 0.5 ms, the droplet array has moved 25 μm and starts oscillating around its new equilibrium position. These underdamped oscillations are caused by the inertia of the droplet array, the spring-like action of the droplet interfaces, and the viscous damping of the fluids. The period of the oscillation is 0.7 ms. Using this value, the mass of the droplet array, and making the approximation that for an underdamped harmonic oscillator the damped and undamped angular frequencies are similar, the horizontal spring constant *k*_x_ can be calculated at 0.21 mN μm^−1^. This value is reasonable, given the forces measured. During actuation, the droplet is distorted by a couple of microns giving an actuating force of 0.69 mN. During a static hold step, the droplet must be distorted approximately half the electrode distance before it fails, giving a blocked force of 2.8 mN. The horizontal spring constant can also be theoretically estimated as 2*γwN*/*D*_f_=0.72 mN μm^−1^, using the surface tension, droplet width, droplet number, and fluid thickness. This estimation is higher than the measured *k*_x_, suggesting that the actuator may not be functioning at its highest potential.

**Figure 8:**
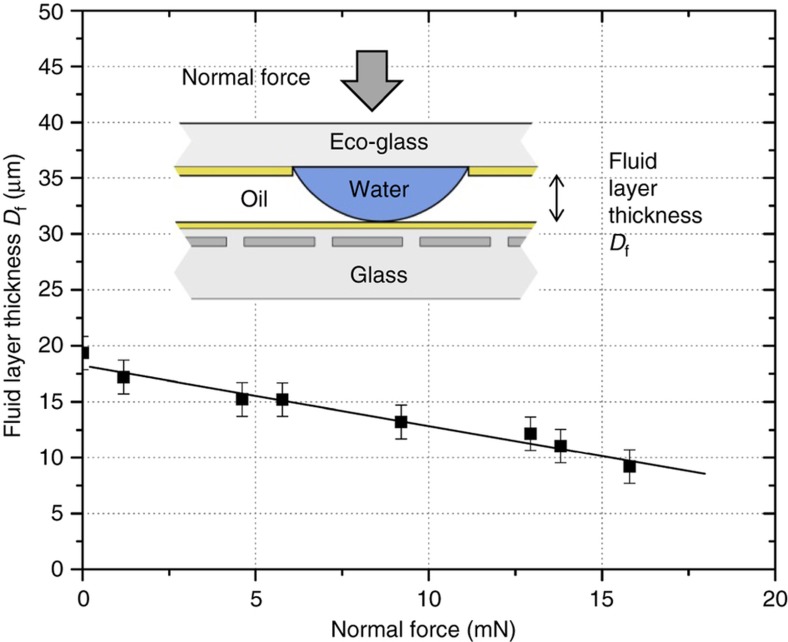
MSA normal pressure response. The separation between the electrode array and the droplet array (*D*_f_), as a function of the force exerted on the top of the droplet array, normal to the array plane, for a 5×5 mm eco-glass array. Force is applied with steel weights while the arrays are horizontal. The droplet array can support over 15 mN of normal force, or >500× its own weight of 26 μN. The slope of the best fit line gives the normal direction spring constant *k*_z_ of 1.9 mN μm^−1^. This measurement is made with no potential applied to the electrodes.

The resistance of the actuator to a force normal to the array plane is also an important characteristic of the system. When enough normal pressure is applied to the top of the droplet array, the droplets become distorted and the actuator will not function. In extreme cases, the droplet array will collapse, squeezing the fluid out from between the arrays. The resilience to a normal force can be measured by looking at how quickly the thickness of the fluid layer decreases as the normal force is increased, as shown in Figure 8 for a glass droplet array. Normal force is simply applied by putting weights on top of the array while it is horizontal. The force and normal displacement are linearly related, again suggesting a spring-like behavior. The spring constant in the normal direction, *k*_z_, can be calculated from the slope of Figure 8 at 1.9 mN μm^−1^. Using *k*_z_, the normal pressure required to compress the drops to half of their height can be calculated at 0.68 kPa. This value correlates well with the internal Laplace pressure of the drops, which is ~1.1 kPa, and their 50% area coverage.

## Discussion

### Scaling

Like other microhydraulic actuators^10^, MSAs improve greatly when scaled. When scaling, we consider reducing all dimensions proportionally except dielectric thickness, which is fixed by the material properties and is already sub-micron. As the actuator is scaled, each droplet interface still contributes a constant force, so reducing droplet pitch increases the force in the array proportionally. Reducing the layer thickness does not affect the actuation force per droplet array area, but does reduce its volume. The combination of these two effects causes the force per cross-sectional area of the MSAs to scale as 1/*D*^2^, where *D* is a characteristic dimension of the actuator. As in other microhydraulic devices^10^, capacitance per volume and frequency of operation both scale as 1/*D*. Therefore, power density also scales as 1/*D*^2^. MSAs should be scalable by another 30× in size and 900× in power density and cross-sectional force before dielectric reliability becomes a limiting factor. Scaling the actuator dimensions has no analog in biology, since biomolecules are fixed in size. Also because the force per drop is linearly related to the surface tension, using mercury instead of water would increase the actuator force by 10×.

### Efficiency

MSAs have efficiency characteristics similar to other simple microhydraulic actuators, which have been measured at up to 68% Ref. (10). Most of the wasted energy is the RC resistive loss. These losses can be as high as 50% when a step function is used to charge and discharge the electrodes. However, charging and discharging the electrodes more carefully, with a triangle wave, for example, or not fully discharging them, leads to lower losses, coming at the expense of power density. Thus, it is possible to trade off efficiency and power through proper biasing.

### Comparison to electrowetting microconveyors

Electrowetting microconveyors^13,14^ have the same principles of operation as the actuators described in this work. They also move a solid platform with attached drops on an electrowetting electrode array. However, important distinctions exist. Microconveyors have been limited to four relatively large semi-spherical water droplets that move along two electrode array rails. MSAs presented here use much smaller semi-cylindrical drops and scale the droplet number and density to provide more force and power. Another important difference from previous microconveyor work is that MSAs have the water phase encapsulated with oil, limiting water evaporation and enabling the use of much smaller droplets. Despite these differences, many of the operational and theoretical principles between MSAs and microconveyors are the same, including scaling. It is helpful to compare the larger scale microconveyors, which have millimeter-size drops, to MSAs, which have 50-μm drops, to validate the scaling theory. While the microconveyor work does not report power density, other figures of merit can be compared. In Moon *et al.*^13^, a microconveyor is used to carry a 180 mg weight, similar to our carrying demonstration of 130 mg. However, the microconveyor uses over 100× the fluidic volume and therefore has a much lower power density, in line with the scaling theory. Geurkens *et al.*^14^ present a detailed measurement of the vertical spring constant for their microconveyor, showing an estimated *k*_z_ of 0.0004 mN μm^−1^, substantially less than our *k*_z_ of 1.9 mN μm^−1^. The difference can again be explained by force and pressure scaling and the difference in the number of drops.

### Comparison to electroactive polymers

Given the structure and operation of our actuators, the comparison to electroactive polymers (EAPs), which have held the title ‘artificial muscles’ for two decades^23^, is inevitable. Microhydraulic stepping actuators function like muscle in a much more fundamental way, but that function itself is not necessarily an operational advantage. A fundamentally important characteristic distinguishes MSAs from EAPs. MSAs can be engineered to charge and discharge an almost arbitrarily large number of times during a single actuation because an actuation consists of many cycles (*N*), and each cycle charges and discharges the entire actuator. In contrast, EAPs charge and discharge only once per actuation. Given that the capacitive energy density of both actuators is ultimately limited by the maximum electric field to roughly the same value, MSAs will have a much higher power density, as they can turn N capacitors worth of energy into actuation power vs. just one capacitor worth for EAPs during each actuation. This type of cyclic capacitive actuation has been demonstrated before in more complex systems such as inchworm MEMS micromotors^6,24^ and electrostatic rotational motors^25^, but MSAs are easier to scale in 3D and arguably easier to make. There are also other characteristics that distinguish MSAs from EAPs, such as lower voltage compared to classical EAPs, no leakage current compared to i-EAPs, and low viscous losses. These advantages may make MSAs usable in a wider variety of applications and microsystems.

### Structure

To fully realize the promise of microhydraulic stepping actuators, both the electrode array and the droplet array should be integrated on a thin substrate. For example, the droplet array can be at the bottom of a thin sheet of polyimide and the electrode array at the top. Then, actuator segments can be stacked into a 3D muscle-like matrix. Both parallel combinations of actuators, which multiply force, and series combinations of actuators, which multiply speed, are possible. The use of laminated ultra-thin glass layers in microhydraulic configurations is also intriguing for optical or display applications where a controllable reshaping of a glass surface could enable new functionality.

### Challenges

Of course, microhydraulic stepping actuators have their own challenges. Being fluidic, critical MSA components can evaporate. Unpackaged, our water/dodecane-based actuators lasted a few hours in a low-humidity atmosphere and a few days in a high-humidity box. Low vapor pressure liquids, such as triethylene glycol and silicone oil, greatly increased lifetime but reduced power due to viscosity and surface tension changes. In the future, a packaging scheme that limits evaporation will be required. Temperature effects are another potential challenge when working with fluids. Freezing can be avoided with the proper selection of fluids down to −20 °C, but temperature effects on surface tension and viscosity must be considered during design. Another challenge is the complexity of fabrication and assembly. Working with thin substrates that are microns or tens of microns thick and fabricating enough area to make an actuator can be challenging. Although we used classical microfabrication techniques, MSAs lend themselves to roll-to-roll processing and imprint lithography. Assembly of the actuator fluids and the setting of droplet pressures requires some degree of self-assembly^26^ and still needs more development, as do techniques to assemble MSAs into appropriate 3D configurations.

## Conclusion

The microhydraulic stepping actuators (MSAs) introduced in this work combine high power density with high actuating efficiency and open a path to new muscle-inspired designs that can power autonomous microsystems. Even the non-optimized and relatively unscaled actuators presented here match the power density of muscle and surpass the metrics of many other types of microactuators. Additionally, MSAs greatly improve in power density when scaled, since the surface tension forces they rely on dominate at smaller dimensions. In the future, 3D arrangements of scaled MSA unit cells could form powerful and efficient micro- and macroactuators. Our single-layer MSAs demonstrate one way in which the internal workings of such a unit cell can be arranged and serve as a step in realizing the potential of microhydraulics.
